# Low intensity near-infrared light promotes bone regeneration via circadian clock protein cryptochrome 1

**DOI:** 10.1038/s41368-022-00207-y

**Published:** 2022-11-14

**Authors:** Jinfeng Peng, Jiajia Zhao, Qingming Tang, Jinyu Wang, Wencheng Song, Xiaofeng Lu, Xiaofei Huang, Guangjin Chen, Wenhao Zheng, Luoying Zhang, Yunyun Han, Chunze Yan, Qian Wan, Lili Chen

**Affiliations:** 1 Department of Stomatology, Union Hospital, Tongji Medical College, Huazhong University of Science and Technology, Wuhan, China; 2 School of Stomatology, Tongji Medical College, Huazhong University of Science and Technology, Wuhan, China; 3 Hubei Province Key Laboratory of Oral and Maxillofacial Development and Regeneration, Wuhan, China; 4 Key Laboratory of Molecular Biophysics of Ministry of Education, College of Life Science and Technology, Huazhong University of Science and Technology, Wuhan, China; 5 Department of Neurobiology, School of Basic Medicine and Tongji Medical College, Huazhong University of Science & Technology, Wuhan, China; 6 State key Laboratory of Materials Processing and Die & Mould Technology, School of Materials Science and Engineering, Huazhong University of Science and Technology, Wuhan, China; 7 Hubei Key Laboratory of Natural Medicinal Chemistry and Resource Evaluation, School of Pharmacy, Huazhong University of Science and Technology, Wuhan, China

**Keywords:** Ubiquitylation, Diseases

## Abstract

Bone regeneration remains a great clinical challenge. Low intensity near-infrared (NIR) light showed strong potential to promote tissue regeneration, offering a promising strategy for bone defect regeneration. However, the effect and underlying mechanism of NIR on bone regeneration remain unclear. We demonstrated that bone regeneration in the rat skull defect model was significantly accelerated with low-intensity NIR stimulation. In vitro studies showed that NIR stimulation could promote the osteoblast differentiation in bone mesenchymal stem cells (BMSCs) and MC3T3-E1 cells, which was associated with increased ubiquitination of the core circadian clock protein Cryptochrome 1 (CRY1) in the nucleus. We found that the reduction of CRY1 induced by NIR light activated the bone morphogenetic protein (BMP) signaling pathways, promoting SMAD1/5/9 phosphorylation and increasing the expression levels of *Runx2* and *Osterix*. NIR light treatment may act through sodium voltage-gated channel *Scn4a*, which may be a potential responder of NIR light to accelerate bone regeneration. Together, these findings suggest that low-intensity NIR light may promote in situ bone regeneration in a CRY1-dependent manner, providing a novel, efficient and non-invasive strategy to promote bone regeneration for clinical bone defects.

## Introduction

Bone defect treatments bring significant medical and socioeconomic challenges involving over two million cases of bone transplant applied nationwide per year.^1,2^ Compared with bone substitutes implantation, in situ bone regeneration can fully regenerate blood vessels and nerves by utilizing endogenous biological resources and reparative capacity,^3^ not limited by donor shortage, immune rejection or postoperative infection,^4–7^ which is an optimal strategy to repair bone defects. However, the repair capability of in situ bone regeneration is still limited, and the speed of bone regeneration usually cannot meet clinical needs.^8,9^ How to improve the efficiency of in situ bone regeneration has become a research hotspot. Driving a sufficient number of target stem cells to ossification at the defect site is the key to in situ bone regeneration.^10^ Previous studies found that low-intensity near-infrared (NIR) light was able to improve local circulation, reduce inflammation and promote cell regeneration,^11–14^ providing a promising strategy to accelerate in situ bone regeneration.

Phototherapy has the advantages of less trauma, high biocompatibility and fast response speed compared with traditional therapeutic strategies.^15,16^ NIR device has been applied to treat alopecia, myopia and dermatosis with good efficacy in clinical practice.^17^ Unlike high-intensity (10-80 W·cm^−2^) and moderate-intensity (up to 1 W/cm^2^) light, mainly used for cauterization and cutting in clinics, low-intensity NIR light (usually below 50 mW·cm^−2^) generally does not cause irreversible damage to biological tissues, but only induces biological responses, promoting the recovery of tissue structures and functions.^18,19^ Low intensity 810 nm NIR light showed not only deep tissue penetration, but also favorable effects on pain relief and bone healing, which was recognized as an appropriate wavelength for treatment of bone defects.^20–23^ It has been reported that low-intensity NIR light therapy can be regulated by nuclear factor kappa-B (NF-κB)/ hypoxia-inducible factor 1-Alpha (HIF-1α) signaling pathway, but mainly during activate neurorehabilitation or angiogenesis.^24,25^ No obvious evidence was found to support its role in the regulation of bone tissues. The underlying mechanism of how NIR light promotes bone regeneration remains to be explored.

Light is the predominant zeitgeber in the circadian rhythm of biological activities.^26^ It has been reported that light can be sensed by local tissues and alter the expression of core circadian clock genes^27,28^ in addition to being detected by the retina to activate the suprachiasmatic nucleus (SCN) and regulate endogenous rhythmic oscillations.^29^ Studies have confirmed that the circadian clock genes play an important role in bone formation and development.^30–32^ Molecules involved in osteoblast differentiation have periodic changes controlled by the circadian system, and genes associated in mineral deposition occur with circadian rhythms.^33,34^ Our previous studies have shown that chondrogenesis and intramembranous ossification are tightly regulated by the circadian clock system.^35–37^ These phenomena suggest that NIR light may have the possibility to influence the circadian clock molecules to regulate bone regeneration.

In this study, we demonstrated that low intensity 810 nm light significantly accelerated bone regeneration in the rat skull defect model. The osteogenic effect of NIR light on bone mesenchymal stem cells (BMSCs) and mouse osteoblastic MC3T3-E1 cells was associated with the ubiquitination of the core circadian clock protein Cryptochrome 1 (CRY1) in the nucleus. Bone morphogenetic protein (BMP) signaling pathways were found involved in the osteogenic differentiation mediated by CRY1. Furthermore, the sodium ion voltage-gated channel *Scn4a* participates in the regulation of NIR light on CRY1, which may be a potential response switch of NIR light for accelerating bone regeneration. Taken together, our study revealed the mechanism of how NIR light promotes bone regeneration through a circadian clock protein, providing new insights to realize a novel, efficient and non-invasive strategy to promote in situ bone regeneration for clinical bone defects.

## Results

### The 810 nm low-intensity NIR light promotes bone regeneration

**Fig. 1:**
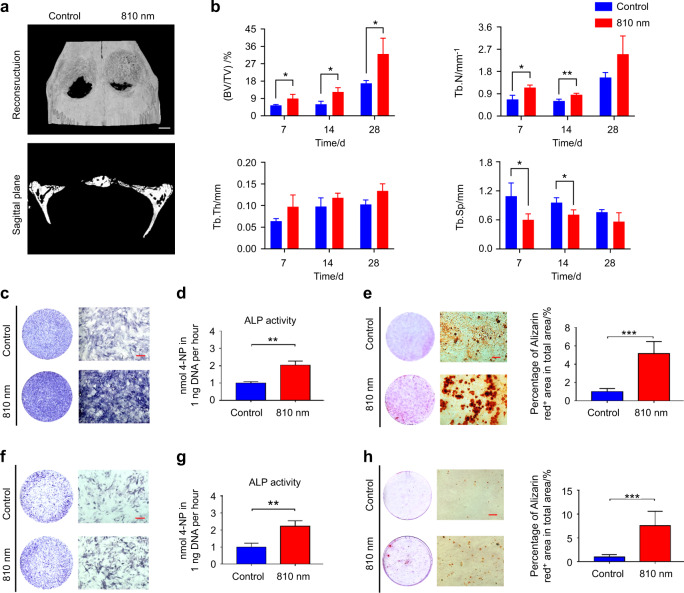
The 810 nm low-intensity NIR light promotes bone regeneration. **a**, **b** Representative images (**a**) of micro-CT reconstruction of skull in rats with or without 810 nm NIR light irradiation at 7, 14, 28 days and analysis (**b**) of bone volume/total volume (BV/TV), trabecular number (Tb.N), trabecular thickness (Tb.Th), trabecular separation (Tb.Sp) of skull defect area. **c**, **d** Representative ALP staining images (**c**) and quantitative detection of ALP activity (**d**) in BMSCs with or without 810 nm NIR light irradiation for 7 days. **e** At 3 weeks after the induction of osteogenic differentiation in BMSCs with or without 810 nm NIR light irradiation, each wells was stained with ARS (left). The ARS-positive areas were quantified from each individual culture plate (right). **f**, **g** Representative ALP staining images (**f**) and quantitative detection of ALP activity (**g**) in MC3T3-E1 cells with or without 810 nm NIR light irradiation for 7 days. **h** At 3 weeks after the induction of osteogenic differentiation in MC3T3-E1 cells with or without 810 nm NIR light irradiation, each wells was stained with ARS (left). The AR-positive areas were quantified from each individual culture plate (right). Data are presented as the mean ± SD. *P < 0.05, **P < 0.01, ***P < 0.001. Scale bar: 1 mm for (**a**) and 100 μm for (**c**, **e**, **f**, **h**)

To determine the effect of 810 nm light on bone defects, we constructed the Sprague Dawley (SD) rat skull defect model and treated the rats with or without 810 nm light (810 ± 15) nm, (25.2 ± 0.1) mW·cm^−2^, Fig. S1a, b). Micro-CT and 3D reconstruction revealed that the skull regeneration was significantly accelerated under the low-intensity NIR light irradiation (Fig. 1a, b). Consistent with the in vivo results, in vitro experiments showed that NIR light could promote osteogenic differentiation of BMSCs and MC3T3-E1 cells. At day 7 of osteogenesis induction, alkaline phosphatase (ALP) expression and activity were significantly increased after treatment with 810 nm light irradiation in both BMSCs and MC3T3-E1 cells (Fig. 1c, d, f, g, respectively). Alizarin red staining images and quantitative analysis indicated that the mineralization level increased about 5.0-fold in the NIR group compared to the control group 14-day after osteogenesis induction (Fig. 1e, h). In these in vitro studies, BMSCs were isolated and identified (Fig. S1c, d). Live/dead staining of BMSCs showed no significant difference in living cell counts 30 min after 810 nm light irradiation with 25 mW·cm^−2^ (Fig. S1e). These data suggest that the 810 nm NIR light has the ability to promote bone regeneration.

### The effect of 810 nm NIR light on osteogenic differentiation was associated with ubiquitination-dependent degradation of CRY1

**Fig. 2:**
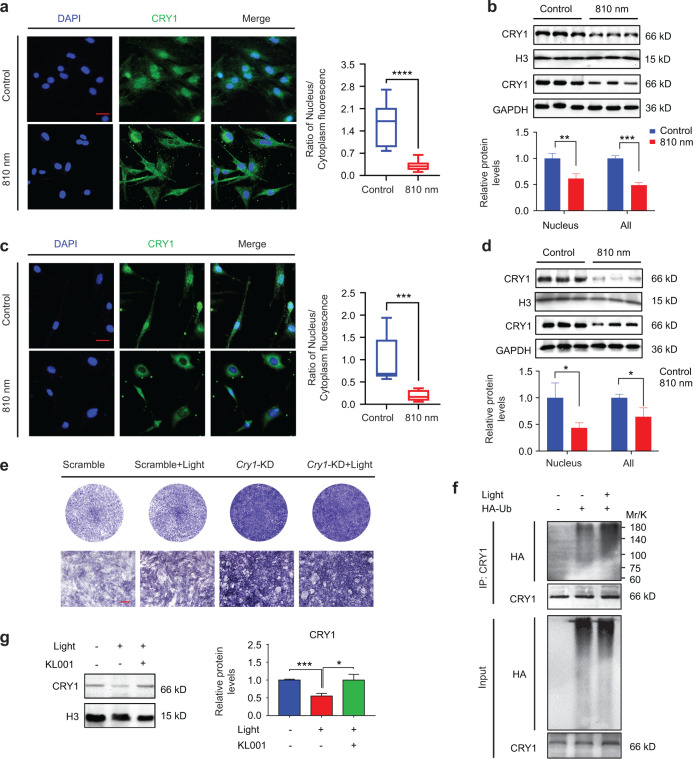
The effect of 810 nm NIR light on osteogenic differentiation was related to the ubiquitination degradation of CRY1. **a** Immunofluorescence staining of CRY1 (green) in BMSCs with or without 810 nm NIR light irradiation, together with nuclei (blue) (left), and the quantitative analysis of nucleus/cytoplasm fluorescence ratio (right) were represented. **b** BMSCs were treated with or without 810 nm NIR light for the indicated minutes. The protein expressions of CRY1 and H3 in the nucleus and the protein expressions of CRY1 and GAPDH in the whole cell were analyzed by Western blot. **c** Immunofluorescence staining of CRY1 (green) in MC3T3-E1 cells with or without 810 nm NIR light irradiation, together with nuclei (blue) (left), and the quantitative analysis of nucleus/cytoplasm fluorescence ratio (right) were represented. **d** MC3T3-E1 cells were treated with or without 810 nm NIR light for the indicated minutes. The protein expressions of CRY1 and H3 in the nucleus and the protein expressions of CRY1 and GAPDH in the whole cell were analyzed by Western blot. **e** Representative ALP staining images in scramble BMSCs and *Cry1*-knockdown BMSCs with or without 810 nm NIR light irradiation for 7 days. **f** MC3T3-E1 cells were transfected with HA-Ub for 24 h and then treated with or without 810 nm NIR light. The ubiquitination level of CRY1 in the nucleus was detected using an anti-HA antibody. **g** BMSCs were treated with or without KL001 (1 μg·mL^−1^) for 2 h before 810 nm light irradiation. The protein expressions of CRY1 and H3 in the nucleus were analyzed by Western blot. Data are presented as the mean ± SD. *P < 0.05, **P < 0.01, ***P < 0.001, ****P < 0.000 1. Scale bar: 50 μm for (**a**, **c**) and 100 μm for (**e**)

To investigate the correlation between the core circadian clock molecules and the regulation of NIR light on osteogenesis, we performed immunofluorescence staining on BMSCs before and after 810 nm light irradiation, and found that the CRY1 protein in the nucleus decreased significantly after NIR irradiation (Fig. 2a). Brain and muscle ARNT-like 1 (BMAL1), Circadian locomotor output cycles kaput (CLOCK), and Cryptochrome 2 (CRY2) showed no significant change between control and light irradiated groups (Fig. S2a, c, d). We then found that the protein level of CRY1 in the nucleus and whole cell extract decreased significantly after NIR irradiation in BMSCs (Fig. 2b), which also was consistent in MC3T3-E1 cells (Fig. 2c, d). The protein level of CRY2 didn’t show significant change in the nucleus (Fig. S2b). To determine whether CRY1 plays an intrinsic role in the osteogenesis of BMSCs under 810 nm light, we performed knockdown and overexpression of *Cry*1 in BMSCs (Fig. S2e, f). There was no significant difference in ALP staining with or without 810 nm light irradiation with *Cry1* knockdown (Fig. 2e). We noticed that the ALP expression was lower in *Cry1*-overexpressed BMSCs, while the NIR light was able to alleviate the osteogenesis inhibition (Fig. S2g). These data indicated that 810 nm low-intensity NIR light was able to enhance osteogenic differentiation by promoting CRY1 reduction in the nucleus. It is reported that CRY1 enters the nucleus to perform its functions after phosphorylation, and subsequently is degraded by a ubiquitin-dependent pathway in the nucleus.^38–40^ To explore how NIR light reduces CRY1 in the nucleus, we detected the phosphorylation level and ubiquitination level of CRY1 before and after light irradiation. The results revealed that the phosphorylation level of CRY1 in the cytoplasm did not decrease, and the ubiquitination level in the nucleus was significantly increased (Fig. 2f, Fig. S2h). We then used KL001, a stabilizer that specifically interacts with CRY and prevents ubiquitin-dependent degradation of CRY,^41^ to test whether the light-induced reduction of CRY1 in the nucleus was associated with ubiquitination-dependent degradation, and found that the reduction of CRY1 disappeared when KL001 was used in both BMSCs and MC3T3-E1 cells (Fig. 2g, Fig. S2i). Our results suggest that 810 nm low-intensity NIR light may promote the ubiquitination-dependent degradation of CRY1 in the nucleus, thus affecting osteogenesis.

### CRY1 reduction activates the BMP signaling pathways to promote osteogenesis

**Fig. 3:**
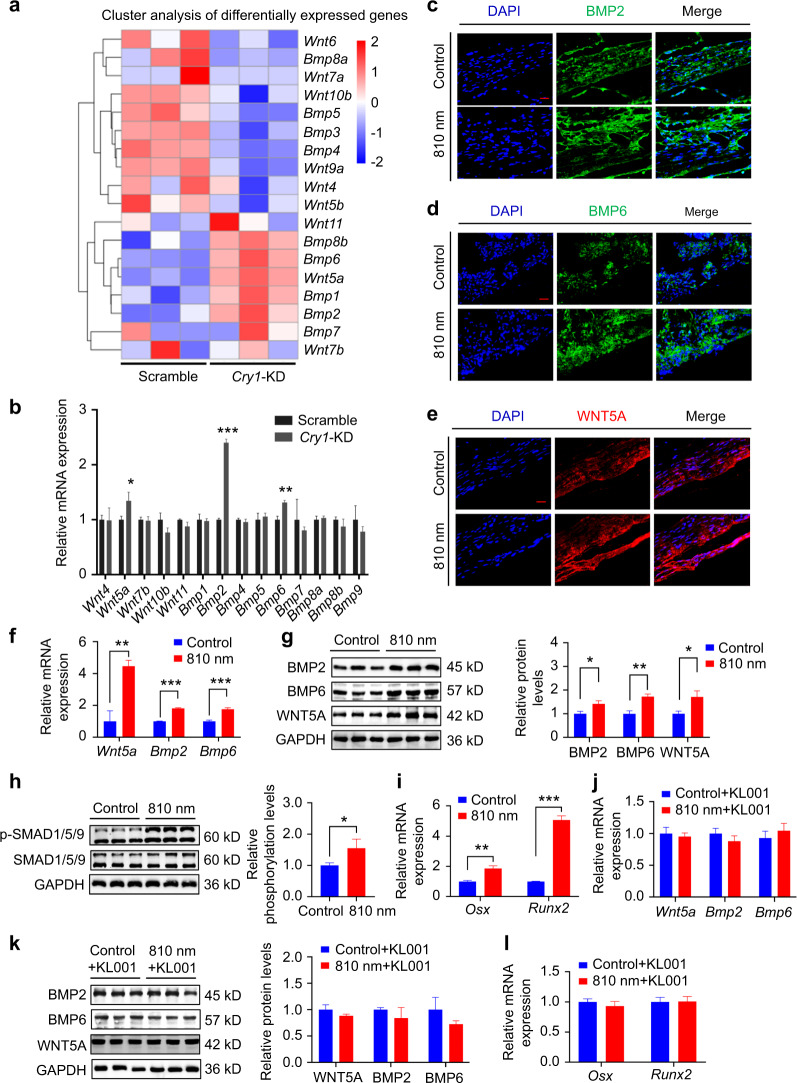
CRY1 reduction activates the BMP signaling pathways to promote osteogenesis. **a** Cluster analysis of differentially expressed genes associated with osteogenesis in scramble BMSCs and *Cry1*-knockdown BMSCs. **b** The transcript levels of differentially expressed genes in (**a**) were determined by qPCR analysis in scramble BMSCs and *Cry1*-knockdown BMSCs. **c**–**e** Immunofluorescence staining of BMP2 (Green), BMP6 (Green) and WNT5A (Red) in skull defect tissues of SD rats at 28 days with or without 810 nm NIR light irradiation, together with nuclei (blue). **f**, **g** At 2 weeks after the induction of osteogenic differentiation in BMSCs with or without 810 nm NIR light irradiation, the mRNA expressions of *Wnt5a*, *Bmp2*, and *Bmp6* and the protein expressions of WNT5A, BMP2, BMP6 and GAPDH were analyzed by qPCR (**f**) and Western blot (**g**). **h** At 2 weeks after the induction of osteogenic differentiation in BMSCs with or without 810 nm NIR light irradiation, the protein expressions of SMAD1/5/9, p-SMAD1/5/9 and GAPDH were analyzed by Western blot (left). Densitometry quantification of p-SMAD1/5/9 compared to SMAD1/5/9 was represented (right). **i** qPCR analysis of the mRNA expressions of *Osx* and *Runx2* in BMSCs treated with or without 810 nm NIR light for 14-day induction of osteogenic differentiation. **j**, **k** BMSCs were treated with or without KL001 (1 μg·mL^−1^) for 2 h before 810 nm light irradiation. The mRNA expressions (**j**) of *Wnt5a*, *Bmp2*, and *Bmp6* were analyzed by qPCR, and the protein expressions (**k**) of WNT5A, BMP2, BMP6 and GAPDH were analyzed by Western blot. **l** qPCR analysis of the mRNA expressions of *Osx* and *Runx2* in BMSCs treated with or without 810 nm NIR light and KL001 (1 μg·mL^−1^) for the indicated time. Data are presented as the mean ± SD. *P < 0.05, **P < 0.01, ***P < 0.001. Scale bar: 20 μm for (**c**–**e**)

To identify the targets of CRY1 independently, we performed genome-wide RNA sequencing (RNA-seq) to acquire the transcriptional profile of *Cry1*-knockdown BMSCs. A total of 1953 genes were found differentially expressed in *Cry1*-knockdown BMSCs. Importantly, many of these genes are osteogenesis-related genes (Fig. 3a). We further used qRT-PCR to validate these differentially expressed osteogenesis-related genes in *Cry1*-knockdown BMSCs, and the results showed that the expression levels of *Bmp2*, *Bmp6*, and *Wnt5a* were upregulated (Fig. 3b). We then performed immunofluorescence staining and found the expression levels of BMP2, BMP6, and Wnt family member 5 A (WNT5A) around bone defect areas were more abundant with NIR light exposure (Fig. 3c–e). qRT-PCR and Western blot showed consistent results (Fig. 3f, g). Together these data suggested that 810 nm low-intensity light can promote the expression of key osteogenic factors including BMP2, BMP6, and WNT5A. The BMP family was reported to play a central role in bone regeneration.^42–44^ As a member of the BMP family, BMP2 and BMP6 regulate bone development through the SMAD signaling pathways.^45–47^ Additionally, the WNT5A signaling pathway is an important component of BMP2-mediated osteogenesis, independent of SMAD phosphorylation dependent pathways.^48^ Therefore, we examined the phosphorylation level of SMAD1/5/9 and the expression levels of *Runx2* and *Osterix (Osx)*, and found they were significantly increased after 810 nm light irradiation (Fig. 3h, i). KL001 was used to determine whether the activation of BMP pathways was associated with NIR-induced ubiquitination-dependent degradation of CRY1, and no changes in the expression levels of BMP2, BMP6, WNT5A, *Runx2*, and *Osx* were observed in KL001 treated BMSCs after light irradiation (Fig. 3j–l). In summary, these results suggest that 810 nm low-intensity NIR light induced reduction of CRY1 may act through the activation of BMP signaling pathways to regulate the osteogenic differentiation of BMSCs.

### The 810 nm NIR light altered the expression of sodium channel *Scn4a*, potassium channel *Kcna6*, and potassium channel *Hcn1*

**Fig. 4:**
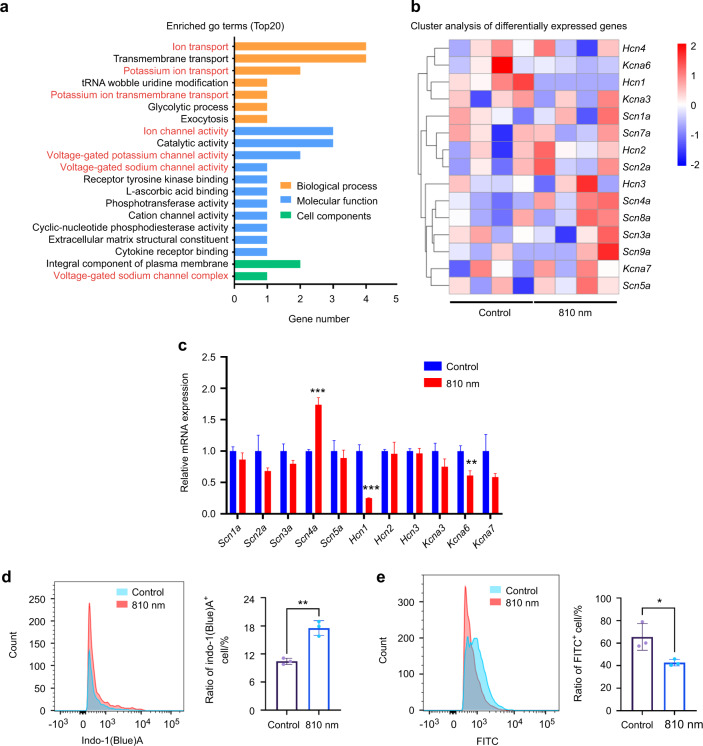
The 810 nm NIR light altered the expression of sodium channel *Scn4a* and potassium channels *Kcna6* and *Hcn1*a. **a** Gene ontology enrichment analysis of the top 20 enriched GO terms in BMSCs with or without 810 nm NIR light irradiation. **b** Cluster analysis of differentially expressed genes associated with potassium and sodium channels in BMSCs with or without 810 nm NIR light irradiation. **c** The transcript levels of differentially expressed genes in (**b**) were determined by qPCR analysis in in BMSCs with or without 810 nm NIR light irradiation. **d**, **e** The concentrations of sodium (**d**) and potassium (**e**) ions in BMSCs were detected by flow cytometry before and after 810 nm NIR light irradiation (left), and the quantitative statistical analysis is represented (right). Data are presented as the mean ± SD. *P < 0.05, **P < 0.01, ***P < 0.001

To identify the molecules that first respond to 810 nm NIR light stimulation, BMSCs were collected immediately after light irradiation and transcriptomic sequencing was performed in the control group and the light irradiated group. The results revealed that 810 nm NIR light mainly affected ion transport, especially sodium and potassium ion voltage-gated channel activity (Fig. 4a, b). We then used qRT-PCR to confirm that the expression of *Sodium voltage-gated channel alpha subunit 4* (*Scn4a*) increased, while the expression of *Potassium voltage-gated channel subfamily a member 6* (*Kcna6*) decreased, and *Hyperpolarization activated cyclic nucleotide gated potassium channel 1* (*Hcn1*) decreased after NIR light irradiation (Fig. 4c). Consistent with the expression data, the intracellular ion concentration changed obviously after light irradiation. The sodium ion concentration increased while the potassium ion concentration decreased (Fig. 4d, e). The above results suggest that 810 nm light has the potential to change the intracellular concentration of sodium ions and potassium ions, which may be associated with the effect of 810 nm NIR light on CRY1.

### The 810 nm light-induced change in sodium channel *Scn4a* was associated with CRY1 reduction and osteogenesis

**Fig. 5:**
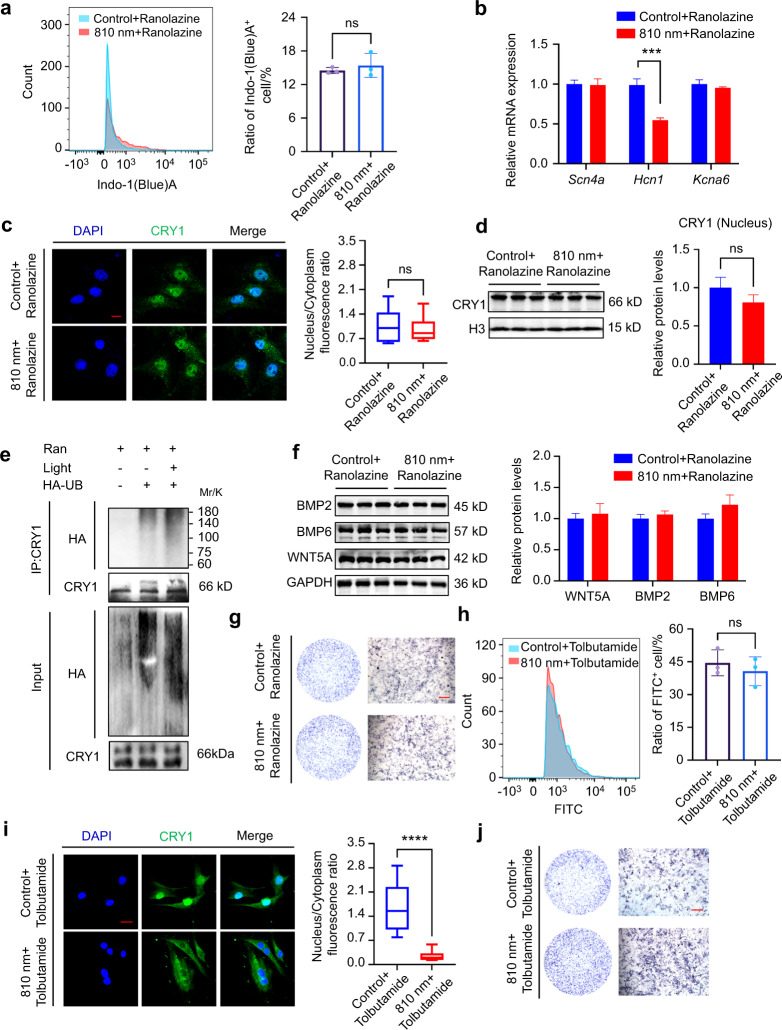
The 810 nm light-induced change in sodium channel *Scn4a* was related to CRY1 reduction and osteogenesis. **a** BMSCs were treated with Ranolazine (30 μmol·L^−1^) for 2 h before 810 nm light irradiation. The concentrations of sodium ions in BMSCs were detected by flow cytometry (left), and the quantitative statistical analysis is represented (right). **b** After treated with Ranolazine (30 μmol·L^−1^) for 2 h, the mRNA expressions of *Scn4a*, *Hcn1*, and *Kcna6* in BMSCs with or without 810 nm NIR light irradiation were determined by qPCR analysis. **c**-**d** BMSCs were treated with Ranolazine (30 μmol·L^−1^) for 2 h. The distribution (CRY1: green, DAPI: blue) (**c**) and the protein expression (**d**) of CRY1 in nucleus with or without 810 nm NIR light irradiation were detected by immunofluorescence staining and Western blot. **e** MC3T3-E1 cells were transfected with HA-Ub for 24 h and then treated with or without 810 nm NIR light. All groups were treated with Ranolazine (Ran) (30 μmol·L^−1^) for 2 h before 810 nm light irradiation. The ubiquitination level of CRY1 in the nucleus was detected using an anti-HA antibody. **f** BMSCs were treated with Ranolazine (30 μmol·L^−1^) for 2 h before 810 nm light irradiation. At 2 weeks after the induction of osteogenic differentiation in BMSCs with or without 810 nm NIR light irradiation, the protein expressions of WNT5A, BMP2, BMP6 and GAPDH were analyzed by Western blot. **g** Representative ALP staining images in 810 nm NIR irradiation BMSCs and no irradiation BMSCs treated with Ranolazine (30 μmol·L^−1^). **h** BMSCs were treated with Tolbutamide (40 μmol·L^−1^) for 2 h before 810 nm light irradiation. The concentrations of potassium in BMSCs were detected by flow cytometry(left), and the quantitative statistical analysis is represented (right). **i** BMSCs were treated with Tolbutamide (40 μmol·L^−1^) for 2 h. Immunofluorescence staining of CRY1 (green) in BMSCs with or without 810 nm NIR light irradiation, together with nuclei (blue) (left), and the quantitative analysis of nucleus/cytoplasm fluorescence ratio (right) were represented. **j** Representative ALP staining images in 810 nm NIR irradiation BMSCs and no irradiation BMSCs treated with Tolbutamide (40 μmol·L^−1^). Data are presented as the mean ± SD. ***P < 0.001, ****P < 0.000 1, ns = not significant. Scale bar: 50 μm for (**c**, **i**) and 100 μm for (**g**, **j**)

To investigate the correlation between 810 nm light-induced alterations in ion channels and CRY1 expression level, we used small molecule compounds Ranolazine or Tolbutamide to inhibit sodium or potassium channels, respectively. We examined the intracellular ion concentrations and confirmed no significant change in the intracellular levels of sodium ions or potassium ions after light exposure combined with the use of Ranolazine or Tolbutamide (Fig. 5a, h). With the treatment of Ranolazine, the expression of *Scn4a* and *Kcna6* showed no significant difference, while the expression of *Hcn1* was still decreased after NIR light irradiation (Fig. 5b). To determine whether *Scn4a* participated in the regulation of NIR light on CRY1, we treated the BMSCs with Ranolazine before light stimulation, and found there was no significant change in the amount of CRY1 in the nucleus in the control and light irradiated groups (Fig. 5c, d). To determine the association of sodium channel *Scn4a* and light-induced ubiquitination of CRY1, we detected the ubiquitination level of CRY1 in MC3T3-E1 cells before and after light irradiation with the use of Ranolazine. The results revealed that the ubiquitination level in the nucleus showed no difference (Fig. 5e). Subsequently, mRNA levels and protein levels of BMP2, BMP6 and WNT5A were measured and there was no significant difference after light exposure when the sodium channel *Scn4a* was blocked (Figs. S3a, Fig. 5f), nor were the expressions of *Osx*, *Runx2*, and ALP (Figs. S3b, Fig. 5g). After treatment of the potassium inhibitor Tolbutamide, *Scn4a* still increased after light irradiation, and CRY1 in the nucleus decreased significantly (Figs. S3c, d, Fig. 5i). We then examined BMP pathway-related osteogenic molecules, and increased expression levels in BMP2, BMP6, WNT5A, SMAD1/5/9 phosphorylation, and ALP were observed in Tolbutamide treated BMSCs after light irradiation (Figs. S3e, f, Fig. 5j). The above results revealed that 810 nm NIR light may promote sodium influx through sodium voltage gated channel S*cn4a*, leading to the reduction of CRY1 in the nucleus, and ultimately accelerate osteogenic differentiation and bone regeneration.

**Fig. 6:**
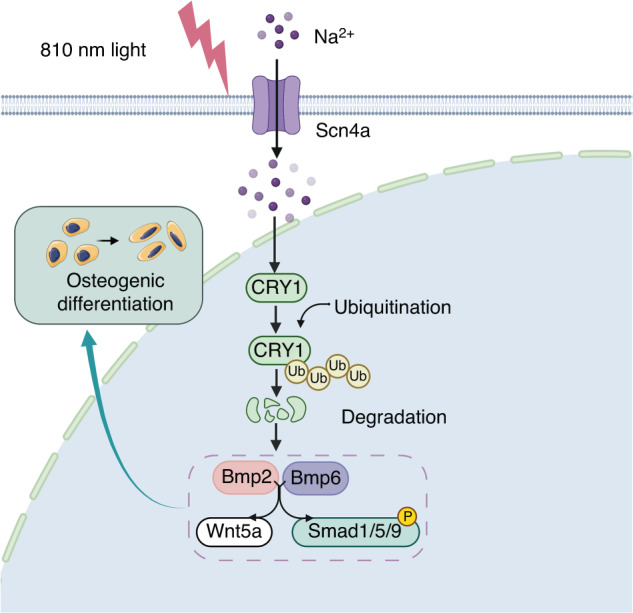
Schematic illustration of the mechanism by which the low-intensity NIR light promotes bone regeneration via CRY1

## Discussion

Bone defects seriously affect quality of life and impact both physical and mental health.^49^ In situ bone regeneration is a relatively safe and ideally optimal repair method, but its efficacy has not been satisfactory. The combined use of mesenchymal stem cells (MSCs) and photobiomodulation (PBM) offers strategies with great therapeutic potential for regenerative medicine.^50^ In this study, we successfully demonstrated the effect of 810 nm low-intensity NIR light on bone regeneration, and elucidated the mechanism by which light activates the BMP signaling pathways by promoting the ubiquitin-dependent degradation of CRY1, providing a novel clinical application of non-invasive NIR treatment in bone defects (Fig. 6).

The infrared light was reported to have thermal effect.^51,52^ In vivo, heat dissipation rate in tissues depends on thermal relaxation time, irradiation time and irradiation density.^53^ We have used the infrared thermal imager to detect the temperature changes during the light irradiation process, and no significant temperature increases were found. This may due to the power densities of NIR light used in the experiments were relatively low (100 mW·cm^−2^ in vivo and 25 mW·cm^−2^ in vitro), which mainly produces photobiomodulation (PBM) effects. PBM is generally considered as a non-thermal response due to low power and short acting time.^54^ Meanwhile, studies involving the use of the thermal effect generated by NIR light usually require the addition of materials with high photothermal conversion efficiency, such as red phosphorus,^55^ indocyanine green (ICG),^56^ and graphene oxide(GO),^57^ which are mostly used for sterilization or tumor cells killing. Low intensity NIR light mainly plays the role of PBM effects, which has the ability to promote tissue regeneration.^8^ Studies have pointed out that light with specific wavelengths can directly affect the function and conformation of photoresponsive receptor proteins or ion channels through photons,^58^ such as channelrhodopsin and rhodopsin,^59,60^ to achieve reversible control of target proteins. Photosensitive ion channels such as photosensitive chlorine ion pump and photosensitive hydrogen ion pump have been used to regulate ion transport across membranes to control cell behaviors.^61,62^

In this study, we first demonstrated that the 810 nm light was able to induce osteogenesis effects by promoting the ubiquitination-dependent degradation of CRY1 in the nucleus. In the transcriptional/translational feedback loop (TTFL) model, CLOCK–BMAL1 heterodimers bind to the E-boxes of *Cry1/2* promoters and activate the transcription of these genes, and increased CRY protein then inhibits its own transcription through binding to CLOCK–BMAL1 complex.^63^ In addition to the TTFL model, F-box and leucine-rich repeat protein 3 (FBXL3) binds to CRY protein in the nucleus and induces ubiquitination-dependent degradation.^64,65^ Glycogen synthase kinase-3 beta (GSK3β)-induced CRY1 phosphorylation potentiates FBXL3-dependent CRY1 degradation in the liver.^66^ In mice, Ser71 and Ser280 of CRY1 are phosphorylation sites of Adenosine 5‘-monophosphate (AMP)-activated protein kinase (AMPK), which can regulate the stability of CRY1, while Ser247 is a phosphorylation site of Mitogen-activated protein kinase (MAPK), which controls CRY1’s role as a transcription inhibitor.^67^ These suggest that the increased level of CRY1 phosphorylation in cytoplasm may also be related to the accelerated ubiquitination-dependent degradation of CRY1. Further studies found that 810 nm low intensity near-infrared light may promote sodium influx by *Scn4a*, thus causing CRY1 reduction and accelerating bone regeneration. This may be related to the intracellular ion concentration, ion signal transduction, and action potential generation. Sodium ion (Na^±^) is the principal extracellular cation and solute, playing an important role in maintaining the osmotic pressure of extracellular fluid, regulating acid-base balance and transmitting nerve signals.^68^ It has been reported that changes in osmotic pressure can lead to changes in the distribution of ubiquitin-proteasome system (UPS).^69^ The change of intracellular sodium concentration will affect the osmotic pressure inside the cell,^70^ which might cause the activation and aggregation of the UPS, leading to increased ubiquitination degradation of CRY1 protein. Studies on the structure of the mammalian CRY1 protein suggest the instability of CRY1.^67,71^ With light stimulating, the action potential and the cell excitability were enhanced, which might directly affect the translation and degradation of intracellular proteins. Furthermore, protein stability is affected by pH value and salt concentration. The increase of intracellular sodium concentration will cause the change of intracellular pH,^72^ which may directly cause conformational changes of CRY1, leading to the exposure of ubiquitination sites and increased degradation. We found that potassium channels play a limited role in light-induced CRY1 reduction and osteogenesis, which may suggest the changes in potassium channels are secondary to sodium channels in response to 810 nm NIR stimulation. Therefore, inhibiting the change of potassium concentration does not completely eliminate the effect of light, and the detailed mechanism still needs to be explored.

CRY proteins play an essential role in regulating mammalian circadian rhythms.^73^ *Cry1* or *Cry2* mutants mice exhibited no rhythmic behavior when kept in constant darkness.^74^ Single *Cry1*-knockout mice showed shorter period than that of wild-type mice.^75^ In addition to regulating circadian behaviors, CRY1 is widely involved in many physiological and behavioral processes such as glucose metabolism, the immune response and bone remodeling.^76–79^ Mice lacking *Crys* display a high bone mass.^80^ Through transcriptome sequencing and functional validation tests, we confirmed that the osteogenesis induced by CRY1 reduction was mediated by BMP signaling pathways. BMP is a member of the Transforming growth factor β (TGF-β) superfamily, playing a critical role in bone regeneration via the classical BMP/SMAD pathway and the non-classical MAPK pathway.^81–83^ Studies reported that the reduction of CRY1 activated the cyclic Adenosine monophosphate (cAMP)/Protein kinase A system (PKA) signaling pathway and the Phosphatidylinositol 3-kinase (PI3K) pathway,^84^ which were able to increase the expression of BMP-related genes and promote fracture healing.^85,86^ Therefore, the regulation of CRY1 on BMP-mediated bone regeneration may also act through cAMP/PKA and PI3K signaling pathways. Moreover, *Cry1* is an important component in the TTFL of biological clock genes, which limits the activity of CLOCK-BMAL1 heterodimer, and thus inhibits the transcription of corresponding downstream genes.^87^ Our previous studies confirmed that BMAL1 was involved in bone development.^35,36^ The BMP pathways were found to be closely related to the circadian clock genes.^88–91^ Reduced CRY1 may also promote osteogenesis by affecting circadian clock genes. Deeper mechanism still needs to be studied and discussed in future scientific research.

There is a desperate need for a more effective and less traumatic way to accelerate bone regeneration. The mild localized NIR light irradiation can achieve non-invasive, remote, and spatiotemporally controlled cell differentiation behaviors, providing a unique strategy for bone tissue regeneration.^92–94^ However, it remains challenging to translate preclinical studies into clinical applications. It is important to fully understand the underlying pathways of light–cellular interactions and to standardize the detailed irradiation parameters to achieve better outcomes. Our study demonstrated a possible target of light-stimulated bone regeneration, providing novel insights into the mechanisms of NIR light assisted osteogenesis and the application of non-invasive phototherapy for clinical bone defects. Further studies are required to identify appropriate light delivery parameters, as well as elucidate the photo-signaling mechanisms involved.

## Materials and methods

### Animals

Male SD rats, aged 7–8 weeks old, weighing 240–260 g were obtained from Beijing HFK Bioscience (Beijing, China) and fed antibiotic-free food and water ad libitum. Animal experiments were performed under a project license (IACUC Number: 2800) approved by the Institutional Animal Care and Use Committee of Tongji Medical College. Anesthesia was achieved by intraperitoneal injection of pentobarbital sodium (40 mg·kg^−1^ of body weight). A linear sagittal incision was made along the midline of calvaria, followed by a full thickness incision to expose periosteum. A round defect (~4 mm in diameter) was drilled on both left and right sides of rat skull using an electric dental bur. A total of 18 rats were randomly grouped. Light irradiation began the day after surgery. All groups had 810 nm near-infrared light irradiation on the right calvarial defect every day, and no light irradiation was performed on the left as control. The rats were sacrificed and their skull were collected at 7 days, 14 days and 28 days after light treatment.

### Micro-computed tomography analysis

The skull specimens were harvested from the rats and fixed in 4% (w/v) paraformaldehyde. Samples were scanned at a resolution of 9 μm using the Skyscan 1176 micro-computed tomography scanner (Bruker, Germany). The reconstructed images were used to generate 3D model of bone structure. Bone mass was evaluated according to BMD, BV/TV, Tb.Th, BS/BV, and trabecular pattern factor (Tb.PF) by the DataViewer software (Inveon Multimodality Scanner, Germany).

### Cell isolation and identification

Healthy SD rats aged 3 weeks with SPF grade were sacrificed by cervical dislocation. Femur and tibia of lower limbs were isolated, and BMSCs were cultured and purified by whole bone marrow adherent method. The growth of primary and passaged cells was monitored by inverted microscope (Nikon, Japan), and surface markers were used for cell identification by a flow cytometer (LSRFortessa™ X-20, BD, U.S.A.). For flow cytometry, cells were harvested and resuspended in ice-cold PBS at a concentration of 1 × 10^6^ cells per mL, and to identify BMSCs, CD90, CD44, and CD29 were used as positive markers, while CD31 and CD45 were used as negative markers (Supplementary Table 3). Cells were cultured in minimum essential medium α (α-MEM; Hyclone, Cytiva Corp., Logan, U.S.A.) supplemented with 10% fetal bovine serum (FBS, Gibco, Invitrogen Corp., California, U.S.A.) and 1% penicillin–streptomycin (Hyclone) in a humidified incubator at 37°C, 5% CO_2_ and passaged to the 3rd generation at a confluency of 70%–80% before use. MC3T3-E1 cells were obtained from the American Type Culture Collection (ATCC).

### Light irradiation

A high uniformity integrated xenon lamp (PLS-FX300HU, Beijing Perfectlight, China) with monochromatic light band-pass filter (*λ* = 810 nm; Beijing Perfectlight, China) was used as the light source. The wavelength of the emitted light was measured by a double beam UV-VIS spectrophotometer (UV-8000A, Shanghai Metash Instruments Co., Ltd., China). In animal experiments, the right defect of each rat skull was irradiated with 810 nm near-infrared light for 10 min daily with an average power density of 100 mW/cm^2^, and the left defect was left without light irradiation as the control group. For in vitro tests, cells were irradiated in six-well plates or 35 mm diameter tissue culture dishes in 1 mL of culture medium in the dark without the lid. The control group was exposed to darkness at the same time without irradiation. Irradiation was performed at a distance of 15 cm, which created a spot size of 16 cm^2^, completely covering the area of the tissue culture plate/dish. Irradiation was performed for 10 min a day with an average power density of 25 mW/cm^2^ measured by an optical power meter (Beijing Perfectlight, China).

### Live/dead cell staining assay

Apoptosis analysis was performed to evaluate the biosafety of light treatment by using Living/Dead cell double staining kit (HR0444, Beijing Biolab Technology Co., Ltd., Beijing, China) followed by confocal imaging. Cells at 1 × 10^4^ were plated in 35 mm confocal dishes. After 810 nm wavelength irradiation, cells were stained by Calcein-AM (1:1 000) for 30 min at room temperature protected from light and washed out with PBS twice. Then, PI (1:20 000) was added to each sample for 5 min in the dark. Images were taken immediately using a confocal laser scanning microscope (Nikon, A1R SI, Japan) with 488 nm and 545 nm laser.

### Cell culture and osteogenic induction

BMSCs and MC3T3-E1 cells were maintained in α-MEM supplemented with 10% FBS and 1% penicillin–streptomycin. The cells were incubated in a humidified incubator with 5% CO_2_ at 37 °C, and changed to fresh medium every 2–3 days. For osteogenic differentiation, cells were seeded at a density of 1 × 10^4^ cells per cm^2^ in α-MEM supplemented with 10% FBS. When the cells were at a confluency of 70%–80%, medium was changed with OriCell^®^ SD rat BMSC osteogenic differentiation medium (RASMX-90021, Cyagen Biosciences Inc., U.S.A.) to induce osteoblast differentiation. Osteogenic differentiation medium was changed every 1–2 days. In addition, a seeding density of 2 × 10^3^ cells per cm^2^ was applied for the fluorescent staining of CRY1.

### Alkaline phosphatase (ALP) and mineralization

Cells were cultured for 7 days in differentiation medium, and the light treatment was repeated once a day for 7 days. ALP activity was measured using the p-nitrophenyl phosphate (Sigma, U.S.A.) method as previously described.^95^ The ALP activity was normalized by DNA content per sample using a QuantiFluor dsDNA System kit (E2670, Promega Corporation, Madison, U.S.A.). For ALP staining, cells were fixed with 4% paraformaldehyde for 30 min and stained with the BCIP/NBT alkaline phosphatase color development kit (C3206, Beyotime, China). For mineralization staining, cells were cultured for 14 days in differentiation medium with or without light irradiation, followed by staining with the Alizarin red S (ARS) staining kit for osteogenesis (C0148S, Beyotime, China). Images were taken using an optical microscope (Nikon, Japan).

### RNA-sequencing and analysis

Total RNAs were extracted from BMSCs of SD rat using TRIzol Reagent (Invitrogen, cat. NO 15596026) following the methods. DNA digestion was carried out after RNA extraction by DNaseI. RNA Integrity was measured by Agilent 2100. Total RNAs at 2 μg were used for stranded RNA sequencing library preparation using Hieff NGS® mRNA Isolation Master Kit (Yeasen, Cat#12603) following the kit instruction. PCR products at 200-500 bp were enriched, quantified and finally sequenced on DNBSEQ-T7 using PE150 model. Raw sequencing data were first filtered by fastp (version 0.36). Low-quality reads were discarded and the reads contaminated with adapter sequences were trimmed. The cleaned data were mapped to the reference genome of *Rattus norvegicus* rn6 from https://www.ncbi.nlm.nih.gov/assembly/GCF_000001895.5 using hisat2 software with default parameters. Gene expression levels were estimated by StringTie for each sample. Genes differentially expressed between groups were identified using DESeq2. SNP and InDel in each sample were detected by samtools and bcftools. A padj cutoff of 0.05 and fold-change cutoff of 2 were used to determine the statistical significance of gene expression differences. Gene ontology (GO) analysis and Kyoto encyclopedia of genes and genomes (KEGG) enrichment analysis for differentially expressed genes were both implemented by clusterProfiler with a padj cutoff of 0.05 to identify statistically significant enrichment.

### Other methods

Immunofluorescence, viral infection, qRT-PCR analysis, western blot analysis, immunoprecipitation and detection of intracellular ion concentration and other methods were described in details in Supplemental Experimental Procedures.

### Statistical analysis

Statistical analyses were performed with GraphPad Prism software version 9.0. All data were presented as mean ± standard deviation (SD). The two-tailed Student’s *t*-test or one-way ANOVA with Tukey’s post hoc test was used to evaluate the statistical significance between groups. *P*-values were considered statistically significant at <0.05.

## Supplementary information

Supplementary materials

## Data Availability

The data generated and/or analyzed during the current study are available from the corresponding author on reasonable request.
