# Supplementary material for: Low intensity near-infrared light promotes bone regeneration via circadian clock protein cryptochrome 1
Source: Int J Oral Sci. 2022 Nov 14;14:53. doi: 10.1038/s41368-022-00207-y (PMC9663728; doi:10.1038/s41368-022-00207-y)
Supplement: Supplementary file 1 — Supplementary materials [file 41368_2022_207_MOESM1_ESM.docx]

*Supplementary Information*

**Low Intensity Near-Infrared Light Promotes Bone Regeneration via Circadian Clock Protein Cryptochrome 1**

Jinfeng Peng ^‡,1,2,3^, Jiajia Zhao^‡,1,2,3^, Qingming Tang^1,2,3^, Jinyu Wang^2,3^, Wencheng Song^1,2,3^, Xiaofeng Lu^1,2,3^, Xiaofei Huang^1,2,3^, Guangjin Chen^1,2,3^, Wenhao Zheng^1,2,3^, Luoying Zhang^4^, Yunyun Han^5^, Chunze Yan^6^, Qian Wan^7^, Lili Chen*^,^^1,2,3^

^1^Department of Stomatology, Union Hospital, Tongji Medical College, Huazhong University of Science and Technology, Wuhan 430022, China

^2^School of Stomatology, Tongji Medical College, Huazhong University of Science and Technology, Wuhan 430030, China

^3^Hubei Province Key Laboratory of Oral and Maxillofacial Development and Regeneration, Wuhan 430022, China

^4^Key Laboratory of Molecular Biophysics of Ministry of Education, College of Life Science and Technology, Huazhong University of Science and Technology, Wuhan 430074, China

^5^Department of Neurobiology, School of Basic Medicine and Tongji Medical College, Huazhong University of Science & Technology, Wuhan 430074, China

^6^State key Laboratory of Materials Processing and Die & Mould Technology, School of Materials Science and Engineering, Huazhong University of Science and Technology, Wuhan 430074, China

^7^Hubei Key Laboratory of Natural Medicinal Chemistry and Resource Evaluation, School of Pharmacy, Huazhong University of Science and Technology, Wuhan 430074, China

‡ Jinfeng Peng and Jiajia Zhao contributed equally to this work.

*Correspondence: Lili Chen (chenlili1030@hust.edu.cn）

***Supplementary Figures***


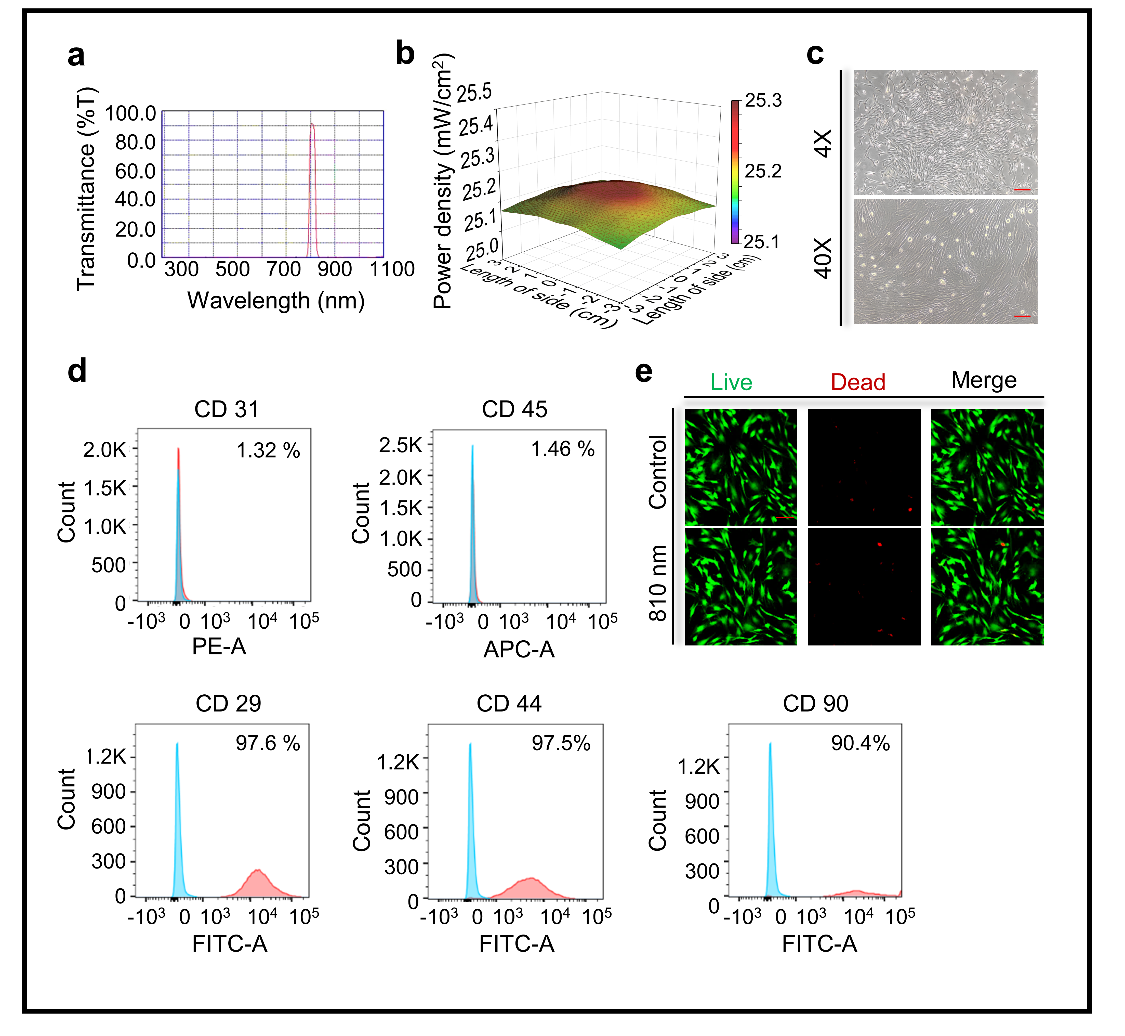


**Fig. S1** Wavelength and biosafety of light, and identification of SD Rat BMSCs. **a**-**b** The wavelength (**a**) and the power density (**b**) of the NIR light. **c** The morphology of the third generation BMSCs isolated and cultured from SD rats. **d** Flow cytometry detection of the surface markers of BMSCs (negative marker: CD31 and CD45; positive marker: CD90, CD44 and CD29). **e** Live/Dead staining of BMSCs treated with or without 810 nm NIR light (25 mW/cm^2^, 30 min) (live cells, green; dead cells, red). Scale bar: 100 μm for upper (**c**) and (**e**), 200 μm for lower (**c**).

**
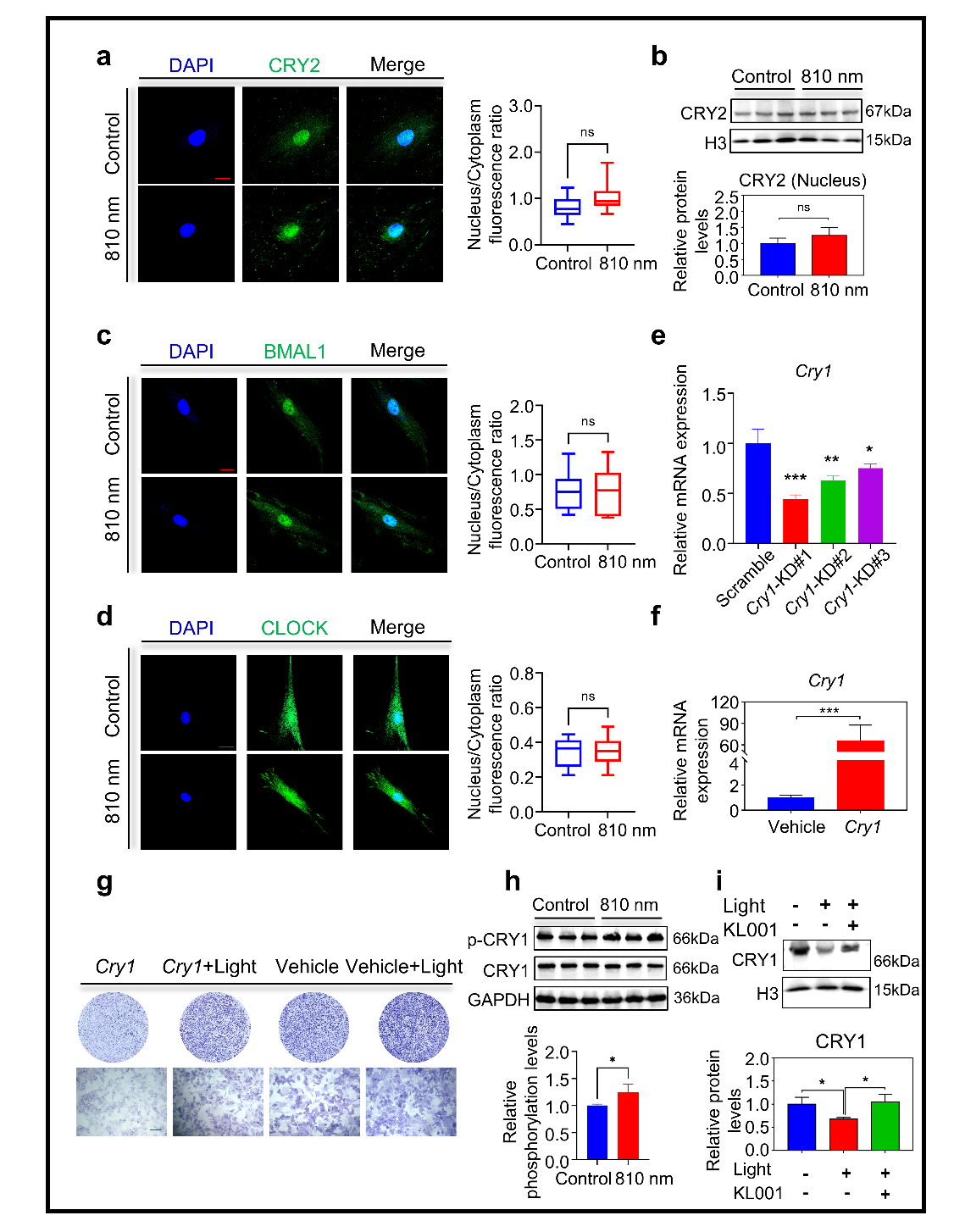
**

**Fig. S2** The effect of 810 nm low intensity NIR light on osteogenic differentiation was related to the ubiquitination degradation of CRY1. **a** Immunofluorescence staining of CRY2 (green) in BMSCs with or without 810 nm NIR light irradiation, and nuclei (blue) (left), and the quantitative analysis of CRY2 nucleus/cytoplasm fluorescence ratio (right) were shown. **b** BMSCs were treated with or without 810 nm NIR light for the indicated minutes. The protein levels of CRY2 and H3 in the nucleus were analyzed by Western blot. **c**-**d** Immunofluorescence staining of BMAL1 (**c**) and CLOCK (**d**) (green) in BMSCs with or without 810 nm NIR light irradiation, and nuclei (blue) (left), and the quantitative analysis of BMAL1 or CLOCK nucleus/cytoplasm fluorescence ratio (right) were shown. **e**-**f** The mRNA expression levels of *Cry1* in scramble BMSCs, *Cry1*-knockdown BMSCs (**e**), vehicle BMSCs, and *Cry1*-overexpressing (*Cry1*) BMSCs (**f**). **g** Representative ALP staining images in vehicle BMSCs and *Cry1*-overexpressing BMSCs with or without 810 nm NIR light irradiation for 7days. **h** BMSCs were treated with or without 810 nm NIR light for the designated times. The protein expression in the cytoplasm of phospho-CRY1 (p-CRY1), CRY1, and GAPDH was analyzed by Western blot (upper). Densitometry quantification of p-CRY1 compared to CRY1 was represented (lower). **i** MC3T3-E1 cells were treated with or without KL001 (1μg/ml) for 2 hours before 810 nm light irradiation. The protein levels of CRY1 and H3 in the nucleus were analyzed by Western blot. Data are presented as mean ± SD. **P* < 0.05, ***P* < 0.01, ****P* <0.001, ns: not significant. Scale bar: 10 μm for (**a**, **c**, **d**) and 100 μm for (**g**).


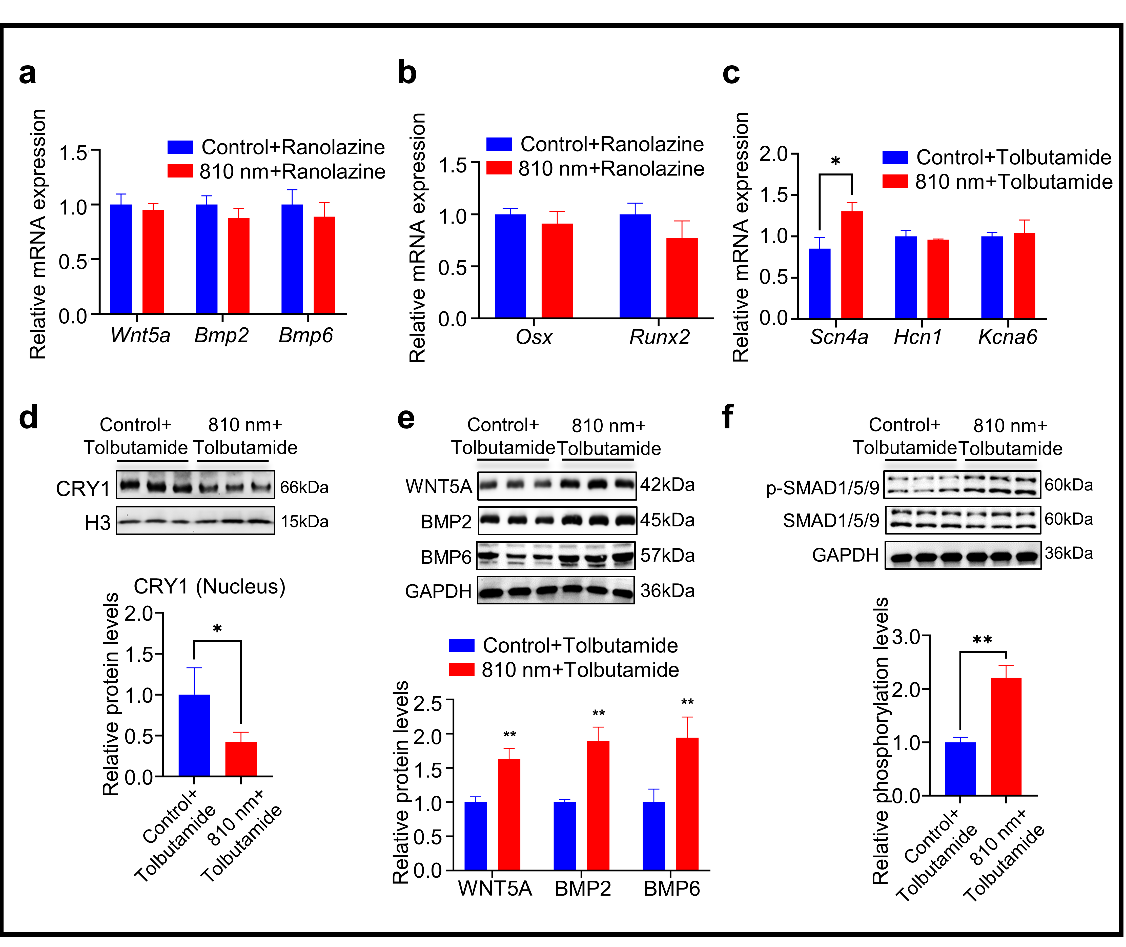


**Fig. S3** Potassium ion channel had no obvious effect on light-induced osteogenesis.

**a** BMSCs were treated with Ranolazine (30 μmol·L^−1^) for 2 hours before 810 nm light irradiation. At 2 weeks after the induction of osteogenic differentiation in BMSCs with or without 810 nm NIR light irradiation, the mRNA expressions of *Wnt5a*, *Bmp2*, and *Bmp6* were analyzed by qPCR. **b** qPCR analysis of the mRNA expressions of *Osx* and *Runx2* in BMSCs with or without 810 nm light irradiation after treated with Ranolazine (30 μmol·L^−1^) for 2 hours. **c** After treated with Tolbutamide (40 μmol·L^−1^) for 2 hours, the mRNA levels of Scn4a, *Hcn1*, and *Kcna6* in BMSCs with or without 810 nm NIR light irradiation were determined by qRT-PCR analysis. **d** BMSCs were treated with Tolbutamide (40 μmol·L^−1^) for 2 hours. The protein expression of CRY1 in nucleus with or without 810 nm NIR light irradiation was detected by Western blot. **e** BMSCs were treated with Tolbutamide (40 μmol·L^−1^) for 2 hours before 810 nm light irradiation. At 2 weeks after the induction of osteogenic differentiation in BMSCs with or without 810 nm NIR light irradiation, the protein levels of WNT5A, BMP2, BMP6 and GAPDH were analyzed by Western blot. **f** BMSCs were treated with Tolbutamide (40 μmol·L^−1^) for 2 hours before 810 nm light irradiation. At 2 weeks after the induction of osteogenic differentiation in BMSCs with or without 810 nm NIR light irradiation, the protein levels of SMAD1/5/9, phospho-SMAD1/5/9 and GAPDH were analyzed by Western blot (left). Densitometry quantification of phospho-SMAD1/5/9 compared to SMAD1/5/9 was represented (right). Data are presented as mean ± SD. **P* < 0.05, ***P* < 0.01.

***Supplementary Tables***

**Supplementary Table 1.** Primer sequences used in qRT-PCR.

| **Primers** | **Sequence (forward/reverse)** | **Usage** |
| --- | --- | --- |
| *r-Gapdh* | 5'-AGGGCTGCCTTCTCTTGTGAC-3'  5'-ATCTCGCTCCTGGAAGATGGTG-3' | qRT-PCR |
| *r-Cry1* | 5'-CAATGCTGGGAGTTGGATGTGG-3'  5'-TTGCAGGGAAGCCTCTTAGGAC-3' | qRT-PCR |
| *r-Wnt4* | 5'-TCGTCTTCGCCGTGTTCT-3'  5'-CTGCACCTGCCTCTGGAT-3' | qRT-PCR |
| *r-Wnt5a* | 5'-GACTTACCTCGGGACTGG-3'  5'-GCGACCTGCTTCATTGAA-3' | qRT-PCR |
| *r-Wnt7b* | 5'-TATGGCATTGACTTTTCC-3'  5'-TAGTACATGAGCCCGACA-3' | qRT-PCR |
| *r-Wnt10b* | 5'-GCCAGGTGGTAACCGAAAA-3'  5'-CTCAGTGCTGCCCCGATG-3' | qRT-PCR |
| *r-Bmp1* | 5'-TCATCCCATTTGTCATCG-3'  5'-GCTTCTCCCAGTGTCTCA-3' | qRT-PCR |
| *r-Bmp2* | 5'-TGGGTTTGTGGTGGAAGTGGC-3'  5'-TGGATGTCCTTTACCGTCGTG-3' | qRT-PCR |
| *r-Bmp4* | 5'-CAAGCGTAGTCCCAAGCA-3'  5'-GTCCCCGTGGCAGTAGAA-3' | qRT-PCR |
| *r-Bmp5* | 5'-TGGGTTCAAGTGGGTTAT-3'  5'-TCTTTCGTGGTTCCGTAG-3' | qRT-PCR |
| *r-Bmp6* | 5'-ATGCCCGGGCTGGGGCGGAGGGCGC-3'  5'-GCGCCCTCCGCCCCAGCCCGGGCAT-3' | qRT-PCR |
| *r-Bmp7* | 5'-AAGACTCCAAAGAACCAAG-3'  5'-CAGTAGTAGGCAGCATAGC-3' | qRT-PCR |
| *r-Bmp8a* | 5'-GCTGCTGAACCATAACAA-3'  5'-GAAGGTGACCATGAAGG-3' | qRT-PCR |
| *r-Bmp8b* | 5'-GAGGTTTGCCGCAGACAC-3'  5'-GCACAGTAATAGGCTGAGTAG-3' | qRT-PCR |
| *r-Bmp9* | 5'-TAGAAGGAAGAGGAGCACT-3'  5'-TTTACACTCGTAGGCATCA-3' | qRT-PCR |
| *r-Osx* | 5'-GCTGCCTACTTACCCGTCT-3'  5'-CCCACTATTGCCAACTGC-3' | qRT-PCR |
| *r-Runx2* | 5'-CAATGCTGGGAGTTGGATGTGG-3'  5'-TTGCAGGGAAGCCTCTTAGGAC-3' | qRT-PCR |
| *r-Scn1a* | 5'-CATCTCCCTCCGCCATTA-3'  5'-CCGCAAGAAACATTCCTACA-3' | qRT-PCR |
| *r-Scn2a* | 5'-GGAGAAACCCCAACTACG-3'  5'-CCAAGAAAATGACCAGCA-3' | qRT-PCR |
| *r-Scn3a* | 5'-TTCCCCAAGACGCAATAG-3'  5'-GTCCTCGAAGGTGCTGTG-3' | qRT-PCR |
| *r-Scn4a* | 5'-GGCTACACCAGCTATGAC-3'  5'-ATGATGACCACGAAGAAG-3' | qRT-PCR |
| *r-Scn5a* | 5'-GATACCGTGTCCCATAGC-3'  5'-AAGATAGTCGTTTCCTCCT-3' | qRT-PCR |
| *r-Hcn1* | 5'-TCATACCAGTTGGAATCAC-3'  5'-ACAGTCCCAGTCCTAAAAT-3' | qRT-PCR |
| *r-Hcn2* | 5'-ATTGAGGACAACACGGAG-3'  5'-GATGACACGAAGTCTACCAC-3' | qRT-PCR |
| *r-Hcn3* | 5'-ATCATCCACCCCTACAGC-3'  5'-GTTCGGAAGTTGAGCACC-3' | qRT-PCR |
| *r-Kcna3* | 5'-TGGTGCGATTCTTTGCTT-3'  5'-GCTGCCCATTACCCTGTC-3' | qRT-PCR |
| *r-Kcna6* | 5'-ATTGCCATCGTCTCAGTG-3'  5'-GGAATCCTCGTCTTCATCT-3' | qRT-PCR |
| *r-Kcna7* | 5'-CCTGGGCACAGAGTTAGC-3'  5'-GCCGATGAAGAGGAAGAAGA-3' | qRT-PCR |

**Supplementary Table 2.** Vectors used in this study.

| **NO.** | **Accession** | **Target or Guide Seq (5’ - 3’)** | **CDS** | **Vectors** | **Vectors ID** |
| --- | --- | --- | --- | --- | --- |
| *Cry1*-RNAi(#1) | NM_198750 | TGCCAGCAGACACCATCACAT | 537..2303 | hU6-MCS-CBh-gcGFP-IRES-puromycin | GV493 |
| *Cry1*-RNAi(#2) | NM_198750 | AGGCACTTACACGTTTGGAAA | 537..2303 | hU6-MCS-CBh-gcGFP-IRES-puromycin | GV493 |
| *Cry1*-RNAi(#3) | NM_198750 | AGGCGTTATTTGCCTGTCCTA | 537..2303 | hU6-MCS-CBh-gcGFP-IRES-puromycin | GV493 |
| Scramble  (*Cry1*) | / | TTCTCCGAACGTGTCACGT | / | hU6-MCS-CBh-gcGFP-IRES-puromycin | GV493 |
| *Cry1* | NM_198750.2 | / |  | pLV[Exp]-Hygro-EF1A>rCry1 | VB180507-1034bpk |
| Vehicle  (*Cry1*) | / | / | / | pLV[Exp]-mPGK>Hygro | VB170720-1013tdj |

**Supplementary Table 3.** Antibodies used in flow cytometry, Western blot, immunofluorescence and immunoprecipitation.

| **Antibodies** | **Cat No.** | **Dilutions** | **Brand** |
| --- | --- | --- | --- |
| Flow cytometry |  |  |  |
| FITC-Anti-CD90 | #202503 | 1:200 | BioLegend |
| APC-Anti-CD45 | #202221 | 1:100 | BioLegend |
| FITC-Anti-CD44 | #202205 | 1:200 | BioLegend |
| FITC-Anti-CD29 | #102205 | 1:50 | BioLegend |
| PE-Anti-CD31 | 555027 | 1:200 | BD |
| Western blot |  |  |  |
| Anti-CRY1 | ab104736 | 1:1000 | Abcam |
| Anti-Phospho-CRY1 | AP1236 | 1:500 | Abclonal |
| Anti-CRY2 | 13997-1-AP | 1:500 | Proteintech |
| Anti-BMP2 | ab14933 | 1:800 | Abcam |
| Anti-BMP6 | A8333 | 1:1000 | Abclonal |
| Anti-WNT5A | A12744 | 1:1000 | Abclonal |
| Anti-SMAD1/SMAD5/SMAD9 | A17439 | 1:1000 | Abclonal |
| Anti-Phospho-SMAD1/SMAD5/SMAD9 | AP0850 | 1:1000 | Abclonal |
| Anti-HA | 51064-2-AP | 1:2000 | Proteintech |
| Anti-H3 | 17168-1-AP | 1:6000 | Proteintech |
| Anti-GAPDH | 10494-1-AP | 1:10000 | Proteintech |
| HRP Goat Anti-Rabbit IgG (H+L) | BL003A | 1:5000 | Biosharp |
| Immunofluorescence |  |  |  |
| Anti-CRY1 | ab104736 | 1:100 | Abcam |
| Anti-CRY2 | 13997-1-AP | 1:100 | Proteintech |
| Anti-BMAL1 | ab93806 | 1:100 | Abcam |
| Anti-CLOCK | ab3517 | 1:100 | Abcam |
| Anti-BMP2 | ab14933 | 1:200 | Abcam |
| Anti-BMP6 | A8333 | 1:200 | Abclonal |
| Anti-WNT5A | A12744 | 1:200 | Abclonal |
| Goat Anti-Rabbit IgG (H+L) (Alexa-Fluor 488) | SA00013-2 | 1:200 | Proteintech |
| Goat Anti-Rabbit IgG (H+L) (Alexa-Fluor Cy3) | SA00009-2 | 1:200 | Proteintech |
| DAPI | BS097 | 1:2500 | Biosharp |
| Immunoprecipitation |  |  |  |
| Anti-CRY1 | ab245564 | 1:100 | Abcam |

***Supplementary Materials and Methods***

**Quantitative real-time polymerase chain reaction (qRT-PCR) analysis**

RNA from cells was extracted using Total RNA extraction reagent (R401-01, Vazyme, China) according to the manual and reverse-transcribed to cDNA using HiScript III RT SuperMix for qPCR (R323-01, Vazyme, China). A 1-μl of cDNA was amplified in a 10-μl volume reaction system using the ChamQ SYBR qPCR Master Mix (Q311-02, Vazyme, China). qPCR was performed using SYBR Green PCR protocol on a real time PCR system (ABI 7300, Applied Biosystems, U.S.A.). Relative mRNA expression levels of target genes were normalized against the mRNA expression level of *Gapdh* and calculated via the 2^-△△Ct^ method. The primers used for amplification are listed in Supplementary Table 1.

**Western blot analysis**

Total protein extracts were obtained from cells utilizing RIPA lysis buffer (P0013B, Beyotime, Shanghai, China) supplemented with fresh protease inhibitors (Invitrogen, U.S.A.) and phosphatase inhibitors (Invitrogen, U.S.A.) and ultrasonic treatment. Proteins in the nucleus were extracted using Nuclear and cytoplasmic protein extraction kit (P0027, Beyotime, Shanghai, China). The concentration of proteins was quantified using the BCA protein assay kit (CW0014S, CWBIO, China). After denaturation by sodium dodecyl sulfatepolyacrylamide gel electrophoresis (SDS-PAGE) loading buffer, proteins were separated via 10% SDS-PAGE and transferred onto 0.45-μm polyvinylidene fluoride (PVDF) membranes. The membranes were then blocked with 5% bovine serum albumin (BSA; A1933, Sigma-Aldrich) for 1 h at room temperature and incubated overnight with primary antibodies (Supplementary Table 3) at 4^◦^C. On the following day, the membranes were washed three times with TBST before reprobed with a secondary goat anti-rabbit antibody for 1 h at room temperature. Next, the membranes were exposed to a Gel imager system (BioSpectrum^®^600, UVP, U.S.A.) to detect reactive signals after incubation with Immobilon western chemiluminescent HRP substrate reagent (WBKLS0500, Millipore, USA). Image J software was used to quantify the band intensity. Relative protein expression levels of the target proteins were normalized against the expression level of GAPDH or H3.

**Immunocytofluorescence staining**

Cells were seeded and cultured in 35 mm confocal dishes. After 30 min of light irradiation, the medium was removed, and cells were washed with PBS, fixed with 4% paraformaldehyde for 10 min, permeated with 0.1% Triton X-100 for 10 min, and blocked with 1% BSA for 30 min at room temperature. Subsequently, the cells were incubated with primary antibodies (Supplementary Table 3) at 4°C overnight. On the next day, the cells were washed with 0.1% PBST (0.1% Tween-20 in PBS) three times and incubated with secondary Alexa-Fluor 488-conjugated goat anti-rabbit IgG(H+L) for 1 h at room temperature in the dark, followed by washing with 0.1% PBST (0.1% Tween-20 in PBS) for three times. Samples were then incubated with DAPI for 10 min. Fluorescent images were captured on a confocal microscope (Nikon, A1R SI, Japan) with NIS software for acquisition.

**Viral infection**

BMSCs were cultivated in 6-well plates and grown to 30% confluence, and then lentivirus transduction was carried out by incubating BMSCs with viral supernatant (MOI = 20) in α-MEM supplemented with 10% FBS containing polybrene (10 µg/mL) for 16 hours in a CO_2_ incubator at 37°. The viral supernatant was replaced with fresh complete medium to recover. After 24 hours, puromycin or hygromycin B was added to select positive clones. The expression levels of target genes were evaluated by qRT-PCR after viral infection. For knockdown of *Cry1*, cells were transduced with recombinant lentivirus particles containing *rCry1* shRNA vector. For overexpression of *Cry1*, BMSCs were transduced with lentivirus containing Lentivirus gene expression vector (3rd generation) inserted with *rCry1* (NM_198750.2) sequence. The sequences used for targeting genes are listed in Supplementary Table 2.

***In vivo* ubiquitination assay**

MC3T3-E1 cells were seeded in 6-well plates at a density of 2×10^4^ cells/cm^2^ overnight and transiently transfected with plasmids expressing HA-tagged ubiquitin for 24 h. Lipo8000^™^ transfection reagent (C0533, Beyotime, China) was used as the delivery system according to the manufacturer’s instructions. Cells were treated with MG132 for 2 hours (HY-13259, MCE) and the irradiated group was exposed to 810 nm light before collection. Cells were washed with PBS and then lysed in cell lysis buffer. The lysates were immunoprecipitated with anti-CRY1 antibody, and then the input and the immunoprecipitated samples were analyzed by Western blotting with anti-HA or anti-CRY1 to detect CRY1 ubiquitination.

**Immunoprecipitation (IP)**

Cells were collected and lysed with Cell lysis buffer for Western and IP (P0013, Beyotime, China) for 40 min on ice. After centrifugation at 12,000 rpm for 5 min, the lysates were then incubated overnight with anti-CRY1 antibody overnight at 4°C, followed by 2-hr incubation with Protein A/G Magnetic Beads (HY-K0202, MCE). The precipitants were then washed four times with lysis buffer, and the immune complexes were boiled with loading buffer at 95℃ for 5 min and analyzed by SDS–PAGE and Western blotting.

**Inhibition of ubiquitination degradation**

KL001 (309928-48-1, Sigma-Aldrich, U.S.A.) was used to inhibit the ubiquitin-dependent degradation of CRY1 protein. BMSCs and MC3T3-E1 cells were seeded in six-well plates at a density of 2 × 10^4^ cells/cm^2^ in α-MEM supplemented with 10% FBS. Before 810 nm light irradiation, KL001 (1μg/ml) was added to medium for 2 hours. After 10 min light irradiation, cells were harvested for western blot and qRT-PCR.

**Histology and immunohistofluorescence staining**

The skull samples of rats were isolated and fixed in 4% paraformaldehyde solution, followed by decalcification with 20% EDTA (ethylenediaminetetraacetic acid) for 4 weeks. Paraffin sections (5 μm thick) were used for histology and immunohistofluorescence staining. The sections were deparaffinized in xylene and hydrated with a graded series of ethanol (100%, 95% and 75% ethanol). For immunohistofluorescence staining, after repaired with high temperature and high pressure, the sections were washed with TBS and blocked with 5% bovine serum albumin. The primary antibodies (Supplementary Table 3) were applied to the slides and incubated at 4 °C overnight. After three washes with TBS, the slides were incubated with Alexa-Fluor 488-conjugated goat anti-rabbit or Alexa-Fluor cy3-conjugated goat anti-rabbit secondary antibody. Nuclei were stained with DAPI. Images were taken using a fluorescence microscope (Nikon, ECLIPSE Ti-SR).

**Inhibition of potassium and sodium channel**

Ranolazine (S1799, Selleck) and Tolbutamide (S2443, Selleck) were used to inhibit the sodium channel and potassium channel, respectively. BMSCs were seeded in six-well plates at a density of 2 × 10^4^ cells/cm^2^ in α-MEM supplemented with 10% FBS. Before 810 nm light irradiation, Ranolazine (30 μM) or Tolbutamide (400 μM) was added to medium for 2 hours. After 10 min light irradiation, the cells were washed twice with sterile PBS and replaced with complete medium. Cells added with the same concentration of DMSO was set as the control group.

**Detection of intracellular ion concentration**

BMSCs were cultured into six-well plates at a density of 2 × 10^4^ cells/cm^2^ in α-MEM supplemented with 10% FBS for 24 h. Then, Ranolazine (30 μM) or Tolbutamide (400 μM) was added and incubated for 2 h. The treated BMSCs were further incubated with 5 µM Na^+^ indicator SBFI AM (sodium-binding benzofuran isophthalate acetoxymethyl ester, MX4509-100UG, MKBio) or K^+^ indicator PBFI-AM (potassium-binding benzofuran isophthalate acetoxymethyl ester, MX4513-100UG, MKBio) in 0.04% Pluronic F-127 (MS4302, MKBio) at 37 °C for 40 min. After 10 min light irradiation, the cells were washed, collected and resuspended with PBS buffer. The fluorescence signal was measured by a flow cytometer (LSRFortessa™ X-20, BD, U.S.A.).
